# Mycophenolic acid induces senescence of vascular precursor cells

**DOI:** 10.1371/journal.pone.0193749

**Published:** 2018-03-14

**Authors:** Ellen Go, Stefan P. Tarnawsky, W. Chris Shelley, Kimihiko Banno, Yang Lin, Chang-Hyun Gil, Emily K. Blue, Laura S. Haneline, Kathleen M. O’Neil, Mervin C. Yoder

**Affiliations:** 1 Division of Pediatric Rheumatology, Riley Hospital for Children at Indiana University Health, Indianapolis, Indiana, United States of America; 2 Department of Pediatrics, Herman B. Wells Center for Pediatric Research, Indiana University School of Medicine, Indianapolis, Indiana, United States of America; Centro Cardiologico Monzino, ITALY

## Abstract

**Objective:**

Endothelial dysfunction is central to the pathogenesis of many rheumatic diseases, typified by vascular inflammation and damage. Immunosuppressive drugs induce disease remission and lead to improved patient survival. However, there remains a higher incidence of cardiovascular disease in these patients even after adequate disease control. The purpose of this study was to determine the effect of mycophenolic acid (MPA), a commonly used immunosuppressive drug in rheumatology, on blood vessel or circulating endothelial colony forming cell number and function.

**Methods:**

We tested whether mycophenolic acid exerts an inhibitory effect on proliferation, clonogenic potential and vasculogenic function of endothelial colony forming cell. We also studied potential mechanisms involved in the observed effects.

**Results:**

Treatment with MPA decreased endothelial colony forming cell proliferation, clonogenic potential and vasculogenic function in a dose-dependent fashion. MPA increased senescence-associated β-galactosidase expression, p21 gene expression and p53 phosphorylation, indicative of activation of cellular senescence. Exogenous guanosine supplementation rescued diminished endothelial colony forming cell proliferation and indices of senescence, consistent with the known mechanism of action of MPA.

**Conclusion:**

Our findings show that clinically relevant doses of MPA have potent anti-angiogenic and pro-senescent effects on vascular precursor cells *in vitro*, thus indicating that treatment with MPA can potentially affect vascular repair and regeneration. This warrants further studies *in vivo* to determine how MPA therapy contributes to vascular dysfunction and increased cardiovascular disease seen in patients with inflammatory rheumatic disease.

## Introduction

Chronic inflammatory rheumatic disease (CIRD) is a heterogeneous group of complex, multisystem disorders characterized by the presence of chronic local or systemic inflammation [1]. CIRD can lead to severe and often life-threatening complications in patients and death if untreated [2–4]. Recently, an increasing prevalence of cardiovascular (CV) morbidity and death associated with CIRD has been recognized [3, 5–7]. The specific cause of the heightened CV risk is unknown, but it has been attributed to the acute and chronic inflammatory state, exposure to traditional cardiac risk factors, earlier diagnosis and initiation of therapy leading to prolonged survival of these patients, and the anti-inflammatory therapies themselves [5–7].

Immunosuppressive agents are the mainstay of therapy and have tremendously improved the outcomes of CIRD patients [8, 9]. Mycophenolic acid (MPA), an inosine monophosphate dehydrogenase (IMPDH) enzyme inhibitor and the active metabolite of mycophenolate mofetil, is an immune suppressive drug that is widely used in treating patients with systemic rheumatic diseases. MPA inhibits guanine nucleotide synthesis that is essential to the survival of lymphocytes known to be involved in the immune response in CIRD [10]. MPA is safer than most immunosuppressive agents and has steroid-sparing effects, both of which are particularly advantageous in the pediatric population. However, MPA has also been reported to restrict proliferation of non-lymphoid cells [11–13].

It is becoming clear that the vascular endothelium in tissues and organs contain endothelial stem and progenitor cells [14–16]. In the human system, both circulating and resident blood vessel progenitor cells have been identified and are called endothelial colony forming cells (ECFC) [17]. ECFC are progenitor cells that exhibit robust proliferative potential, clonogenic properties, and unique vasculogenic function capable of forming new vessels *in vivo* that become part of the systemic circulation of the host [18, 19]. The frequent use of MPA in treating diseases associated with an increased risk for developing vascular dysfunction and CV complications, raises the question as to the effects of MPA on ECFC number and function. We hypothesized that MPA diminishes the proliferative potential and vasculogenic function of human ECFC.

## Materials and methods

### Isolation and culture of human umbilical cord derived ECFC

Human umbilical cord blood samples from healthy term newborns were collected in CPD solution and processed as preciously described [18]. The Institutional Review Board at the Indiana University School of Medicine approved all protocols. Informed consent was waived by the ethics committee. In brief, blood was diluted 1:1 with Hanks balanced salt solution, layered over Histopaque 1077, centrifuged and washed with complete EGM-2 medium (EBM-2 [Cambrex, Walkersville, MD] supplemented with 10% fetal bovine serum [Hyclone, Logan, UT], 2% penicillin/streptomycin and 0.25 μg/mL amphotericin B) to isolate mononuclear cells (MNC). MNC were re-suspended in EGM-2 medium and seeded onto six well plates pre-coated with type I rat tail collagen (BD Biosciences, Bedford, MA) and cultured in a 37 °C with 5% CO_2_ humidified incubator for 24 hours. Medium was changed daily for seven days and then every other day until the first passage. Once confluent, cells were detached with TrypLE^™^ Express (Gibco), counted and either plated onto 75-cm^2^ tissue culture flasks pre-coated with type 1 rat tail collagen for further passage or frozen at a density of 1x10^6^ cells/ml at -80 °C for future use.

**Fig 1:**
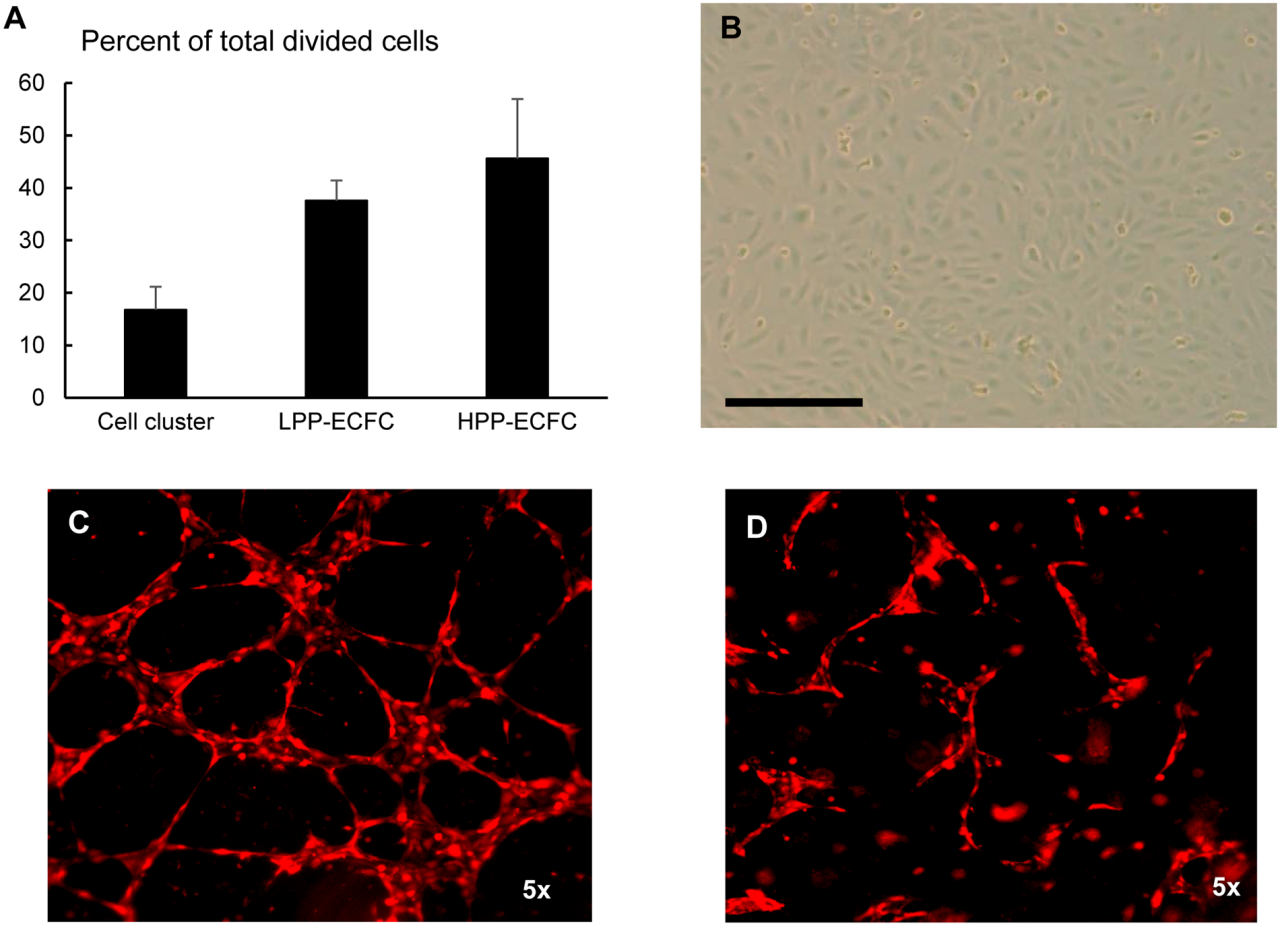
ECFC identification criteria. ECFC display clonogenic potential (A), cobblestone morphology [scale bar = 100 μm] (B) and have ability to form cord-like structures in 2D Matrigel dishes (C) and vessel-like structures in 3D collagen matrix (D) in vitro. Abbreviations: ECFC = endothelial colony forming cell, MPA = mycophenolic acid, LPP-ECFC = low proliferative potential—endothelial colony forming cells, HPP-ECFC = high proliferative potential-endothelial colony forming cells.

ECFC appear as individual colonies that proliferate to form a monolayer of adherent cells with cobblestone morphology. They display endothelial properties, exhibit clonogenic potential, and ability to form cord-like structures in 2D Matrigel coated dishes and tube-like structures de novo from a single cell suspension in 3D collagen matrix in vitro (Fig 1). Previous work demonstrated that ECFC are characterized by the expression of CD31, CD34, KDR, CD144, CD105, CD146 and vWF cell surface proteins and absence of hematopoietic cell markers CD45, CD14, CD115 and AC133 [18].

### Mycophenolic acid

Mycophenolate stock solution was made by dissolving mycophenolic acid powder (C_17_H_20_O_6_, MW: 320.3; Sigma-Aldrich) in 0.6% methanol before diluting in culture medium to the desired final concentrations.

### Cell proliferation assay

#### a. Cell counting by Trypan blue exclusion

Equal densities (1x10^4^ cells) of ECFC were seeded onto 24-well culture plate wells and exposed to EGM-2 medium with or without MPA at 0.1, 1, 2.5 and 5 μM concentrations. Cells were collected and an aliquot was counted on a haemocytometer in the presence of Trypan blue dye after 24, 48 and 72 hours of incubation. Viable cells were identified as Trypan excluding cells.

#### b. Single cell assay for clonal proliferative potential

Single endothelial cells (EC) were sorted using a FACSAria Sorter (Becton Dickinson, San Jose) and placed into each well with complete EGM-2 medium in a 96-well tissue culture plate pre-coated with collagen. Wells were examined the day after plating to ensure presence of a single cell per well. Culture medium was replaced with complete EGM-2 medium with or without MPA at various concentrations, and then incubated at 37 °C with 5% CO_2_ for 13 days with two media changes. On day 14, cells were fixed and stained with 4',6-diamidino-2-phenylindole stain (Sigma-Aldrich). Each well was then examined for colony formation using a Zeiss Axiovert 25 CFL inverted microscope with a 10× CP-ACHROMAT/0.12 NA objective. For quantitative analysis, wells that had ≥2 EC were scored and examined further by visual counting and classified as endothelial cell clusters (2–50 cells), low proliferative potential (LPP)-ECFCs (51–2,000 cells), and high proliferative potential (HPP)-ECFCs (≥2,000 cells per colony).

#### c. Carboxyfluorescein succinimidyl ester (CFSE)

CFSE (Molecular Probes, Eugene, OR) was used to quantify human ECFC proliferation. Cells were stained with CFSE according to manufacturer instructions. 7.5-15x10^4^ CFSE-stained cells were cultured in complete EGM-2 medium at defined concentrations of MPA at 0, 1, 3, 5 and 7 days. After appropriate time of culture, cells were harvested fixed with 4% formaldehyde, and stored at 4 °C. On day 7, the CFSE mean fluorescence intensity (MFI) of all harvested samples was measured using flow cytometry (BD FACS Canto II) and analyzed using FlowJo software (TreeStar). The data represent averages of 4 biological replicates. To account for differences in CFSE staining between replicates, the MFI of the day 0 sample was used as an internal control. Thereby, the MFI of each sample was normalized by dividing it by the MFI of the day 0 control. Results are plotted on a log2 scale in order to visualize the doubling nature of cell proliferation.

### *In vitro* capillary network formation

#### a. Two-dimensional (2D) Matrigel assay

1x10^4^ cells per well were plated in triplicate onto 96-well plates coated with 50 μL of growth-factor reduced Matrigel (BD Bioscience). Complete EGM-2 medium with or without MPA were added to the well and incubated at 37 °C with 5% CO_2_ for 6 hours. Three images were taken from each well at 10x magnification using a Zeiss Axiovert 25 CFL inverted microscope with a 10x CP-ACHROMAT/0.12 NA objective (S1 Fig). Total cord length and average cord area were calculated using ImageJ software (NCBI).

#### b. Three-dimensional (3D) collagen assay

Reagents for 3D collagen matrix were purchased from GeniPhys (Zionsville, IN) unless otherwise specified. To make the matrix, stock oligomer was diluted in 0.01 N hydrogen chloride (HCl) and neutralized according to the manufacturer’s recommendations to achieve a final oligomer concentration of 1.56 mg/ml (250 Pa matrix stiffness) before adding 10% human platelet lysate (SALK, Graz, Austria). Matrix was maintained in solution at 4 °C until used. 1x10^5^ ECFCs (60 μL) were suspended in the collagen matrix. The collagen-cell suspensions were plated onto 96-well culture plate wells and allowed to polymerize at 37 °C for 30 minutes before covering with MPA treated and untreated culture medium. Cells were incubated at 37 °C with 5% CO_2_ for 3 days with daily media change. After three days, cells were fixed and stained with 0.1% toluidine blue O dye. Three images were taken at a uniform depth from the surface of the matrix using a Zeiss Axiovert 25 CFL inverted microscope (S1 Fig). Average cord area was quantified using ImageJ software (NCBI).

### *In vitro* scratch assay

4x10^4^ cells per well were plated in triplicate onto a 12-well plate and cultured in complete EGM-2 medium. Upon reaching 70–80% confluence, a single straight line through the cell monolayer was made using a sterile p1000 pipette tip. Cells were washed once with PBS to remove cell debris and replaced with medium with or without MPA. Images were taken at various time points after the scratch and measured the average distance between the lines after 15 hours. We used image J software (NCBI) to quantify gap distance.

### Apoptosis

The effect of MPA on ECFC death by apoptosis was examined using annexin V-fluorescein isothiocyanate (FITC) and propidium iodide (PI) analysis by flow cytometry. 5-35x10^4^ cells were plated and treated with varying concentrations of MPA for 7 days with one media change. Cells were collected and incubated for 15 minutes at room temperature with annexin V-FITC (BD Pharmigen) and PI (BD Pharmigen) in the dark, and immediately analyzed on a flow cytometer (BD Biosciences San Jose, CA). Data were processed using FlowJo software (TreeStar), determining the percentage of apoptotic cells.

### Senescence associated beta-galactosidase (SA-beta-gal) staining

To determine MPA effect on cellular senescence, SA-beta-gal assay was performed using a Senescence Detection kit (BioVision, Milpitas, CA) according to the manufacturer’s instructions. Briefly, cells were washed with PBS, fixed using fixing solution for 10–15 minutes, washed again with PBS, stained with SA-beta-gal solution overnight at 37 °C and imaged under bright-field microscope. To quantify SA-beta-gal activity, the percent of positively stained blue cells versus total cells were counted (500–600 cells).

### Cell cycle analysis

ECFC (3-5x10^5^) were exposed to either vehicle control or MPA 1 μM. After 3 days, cells were treated with 5’-bromo-2’-deoxyuridine (BrdU) labeling reagent (Invitrogen) for 1 hour at 37 °C with 5% CO2. Cells were stained using Alexa Flour 488 mouse anti-BrdU (Invitrogen) for 90 minutes at room temperature and 7-AAD (Life Technologies) for 15 minutes at room temperature. Samples were analyzed by flow cytometry on the LSRII 407nm laser and a minimum of 10,000 events was recorded per sample. Analysis was performed using FlowJo Single Cell Analysis Software vX.0.6.

ECFC (5-50x10^3^) cells were seeded in 10 cm^2^ culture dish and incubated in EGM-2 medium with and without MPA for either 3 or 7 days. Cells were collected, washed, fixed with 3.7% formaldehyde for 10 minutes at room temperature, washed again, and re-suspended in 25 μg/ml PI solution (Sigma Aldrich) for 15 minutes in the dark. The percentage of cells at the G_0_/G_1_, S and G_2_/M phases of the cell cycle were analyzed on a flow cytometry system (BD Biosciences, San Jose, CA) and FlowJo software (TreeStar).

### RNA isolation and real-time quantitative PCR

Total mRNA was extracted from the cell using RNAeasy Micro Kit (Qiagen) per the manufacturer’s instructions. The concentration and purity of the extracted RNA was measured using a Nanodrop 2000 (Thermo Scientific, Wilmington, DE). cDNA was synthesized from 500 μg of total RNA using SuperScript^™^ III First-Strand Synthesis System (Invitrogen) with Oligo-dT primers per the manufacturer’s instructions, and used as a template for PCR amplification performed on a LifeEco thermal cycler. qPCR analysis was performed using SYBR Green PCR Master Mix (Applied Biosystems, UK) and real time detection on 7500 Real-Time PCR machine (Applied Biosystems, UK). Relative gene expression was calculated using the 2^−ΔΔCt^ method normalized to beta-2-microglobulin mRNA expression levels. The primers used are listed in supplementary information online (S1 Table).

### ELISA

p53 (Total/Phospho) InstantOne ELISA Kit (Affymetrix) was used to specifically detect endogenous levels of total (tot) and phosphorylated (ph) p53 protein generation per the manufacturer’s instructions. Briefly, cells were lysed with lysis buffer mix. Total protein concentration was determined by bicinchoninic acid (BCA) method. 0.2–0.3 mg/ml protein lysates were added to each assay well followed by prepared tot-p53 and ph-p53 antibody cocktail (capture antibody and detection antibody regents). After 1 hour incubation at 300 rpm, wells were washed 3x before adding detection reagent. The reaction was terminated with stop solution after 15 minutes incubation with shaking at 300rpm and the absorbance immediately measured spectrophotometrically at 450 nm.

### Statistical analysis

All experiments were performed ≥ three times with 2–3 technical replicates for each assay. Data are presented as the means ± SD. Two-tailed Student’s *t*-test or Fisher’s exact test were used to assess for significance of differences between two groups.

## Results

### MPA inhibits ECFC proliferation

**Fig 2:**
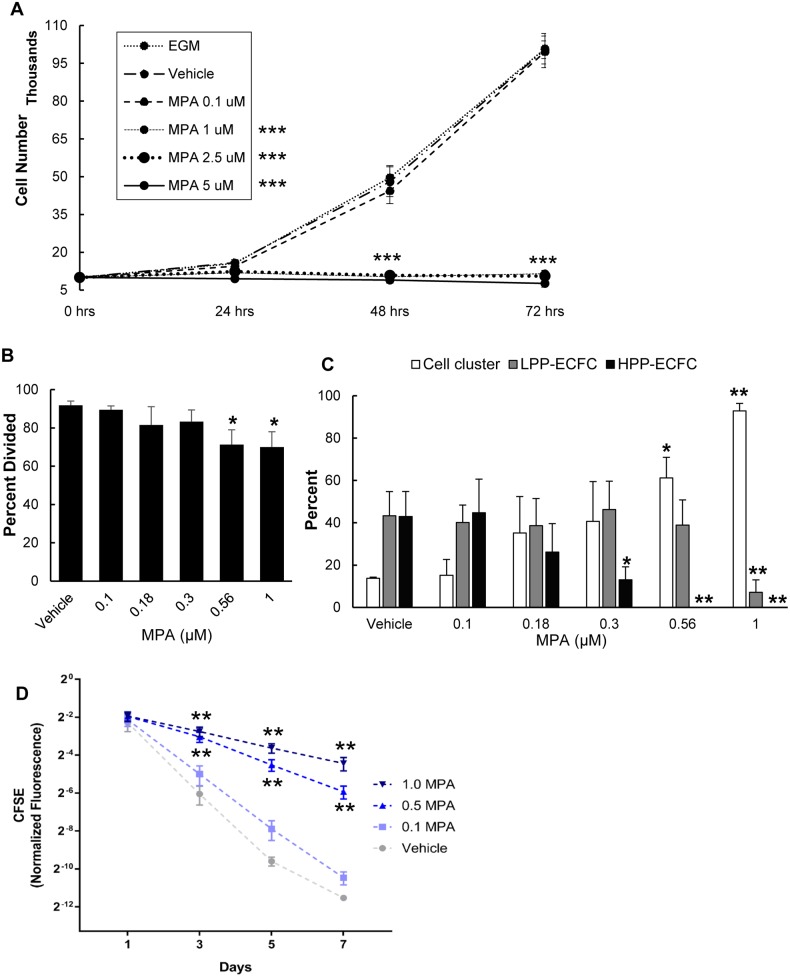
MPA inhibits cell proliferation in a dose dependent manner. (A) Cell growth curve was evaluated using trypan blue staining to measure viable cells at 24, 48 and 72 hours. 1–5 μM concentration of MPA inhibited cell proliferation (n = 3). (B) Percentage of single cord blood-derived ECFC undergoing at least one cell division 14 days after MPA treatment (n = 3). (C) Percentage of cell clusters, LPP-ECFCs and HPP-ECFC 14 days after MPA treatment using single cell analysis (n = 3). (D) Proliferation of ECFC in the absence or presence of MPA (0.1, 0.5 and 1 μM) measured after 1, 3, 5 and 7 days by a FACS-based CFSE dilution assay. While 0.1 μM MPA did not impact ECFC division, MPA at higher concentrations significantly diminished ECFC division (n = 4). Results represent the mean ± SD. *P <0.05, **P<0.005, *** P<0.001 compared to vehicle. Abbreviations: ECFC = endothelial colony forming cell, EGM = endothelial growth medium, MPA = mycophenolic acid, LPP-ECFC = low proliferative potential—endothelial colony forming cells, HPP-ECFC = high proliferative potential-endothelial colony forming cells, CFSE = carboxyfluorescein succinimidyl ester.

To determine whether endothelial cells are affected by MPA, human cord blood derived ECFCs were cultured with increasing MPA concentrations through the usual patient targeted therapeutic range and examined for cell proliferation. The cell number increased at least 4-fold after 48 hours and 10-fold after 72 hours of culture in untreated control cells and cells exposed to 0.1 μM MPA concentration. There was no significant change in viable cell counts of ECFC cultured in 1, 2.5 and 5 μM MPA concentrations at either time (Fig 2A), suggesting a complete block in cell proliferation. ECFC display a hierarchy of proliferative potential that can be discriminated on a single-cell level [18]. Single cell assay was performed to evaluate the effect of MPA on ECFC clonogenic potential. MPA concentrations used (0.1–1 μM) were selected based on results from the cell proliferation assay performed above (Fig 2A). At 0.1 μM MPA concentration, 89.5% (±2.0%, NS) of the single endothelial cells underwent ≥1 cell division. We observed that 40.1% (±8.2%, NS) of the total cells that divided had formed colonies of 51–2,000 cells per well (LPP-ECFC) and 55.7% (±16%, NS) formed >2,000 cells (HPP-ECFC that display self-renewal potential). At 1 μM MPA concentration, 70% (±8%, p<0.05) of the single cells plated divided and the majority of cells were restricted to forming small EC clusters (92.9%±3.5%, p<0.005). No HPP-ECFCs were seen to emerge from either 0.56 μM or 1 μM MPA treated cells. MPA treatment resulted in a dose-dependent decrease in overall colony formation (Fig 2B and 2C). The loss of HPP-ECFC at the higher doses suggests that the most primitive cells are more susceptible to MPA effects. ECFC proliferation was measured using CFSE staining. The CFSE dye stains intracellular proteins and is evenly distributed in dividing progeny cells after cellular division. As such, the rate of decrease of CFSE MFI among cultured cells is proportional to their rate of proliferation. We used flow cytometry to measure the MFI of CFSE-stained ECFC during 7 days of culture at increasing doses of MPA. As expected, cells cultured with vehicle or 0.1 μM MPA regularly divided and displayed diminished MFI over the 7 days culture. We observed significantly greater CFSE MFI retention (indicative of lack of cell division) in ECFC treated with MPA 0.5 and 1 μM MPA compared with ECFC treated with vehicle control (Fig 2D). These results indicate that ECFC treated with MPA displayed significantly decreased proliferation, consistent with the clonogenic data above (Fig 2C).

### MPA delays ECFC migration

**Fig 3:**
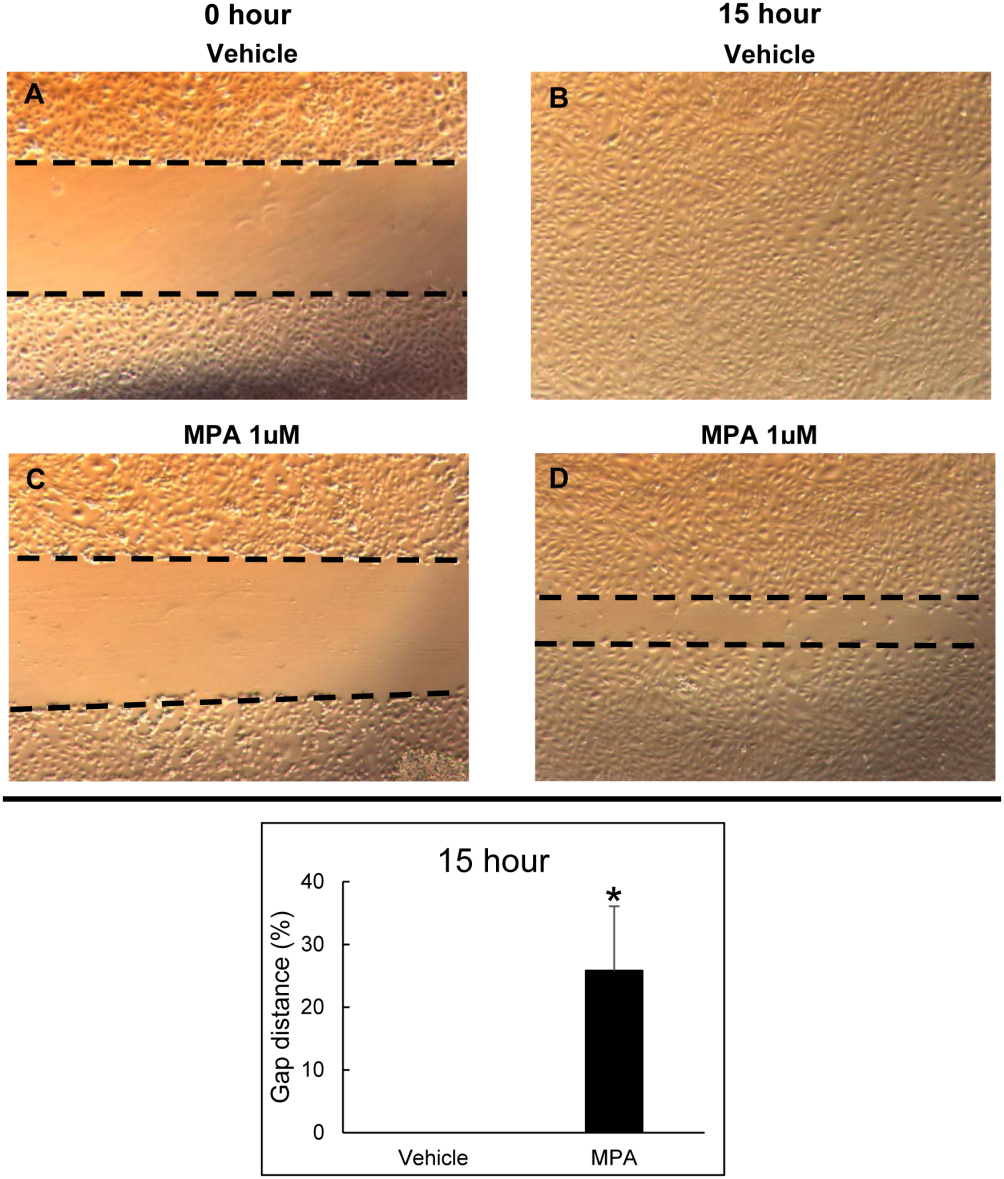
MPA treatment impairs ECFC migration. Cell migration was assessed using an *in vitro* scratch assay with and without MPA treatment. Upper panel: Representative phase-contrast images of ECFC incubated with vehicle control media at time 0 hour after wounding (A) and closure of the gap after 15 hours (B). ECFC incubated with 1 μM MPA at time 0 hour after wounding (C) and presence of a gap after 15 hours (D). Lower panel: Quantification of gap distance after 15 hours of incubation. Gap fully closed in vehicle treated wells while a gap is still clearly visible in cells treated with 1 μM MPA (25.8% ± 10%, *p<0.005) compared to vehicle (n = 4 to 6) Abbreviations: MPA = mycophenolic acid, ECFC = endothelial colony forming cells.

EC migration into a denuded vessel is a vital process in wound healing. To determine whether MPA inhibits migration of ECFC, we performed an *in vitro* scratch assay. A line was drawn along the leading edges of each cell front at time 0 hour and 15 hour and measured the gap distance as percentage of un-occupied area at 15 hour divided by 0 hour exposure to media with and without treatment. Untreated cells completely closed the gap when examined after 15 hours, but a cell-free area is clearly visible in the MPA-treated (1 μM) cells (Fig 3). The lag in gap closure observed after drug treatment can be attributed to delayed cell migration.

### MPA reduces ECFC function

**Fig 4:**
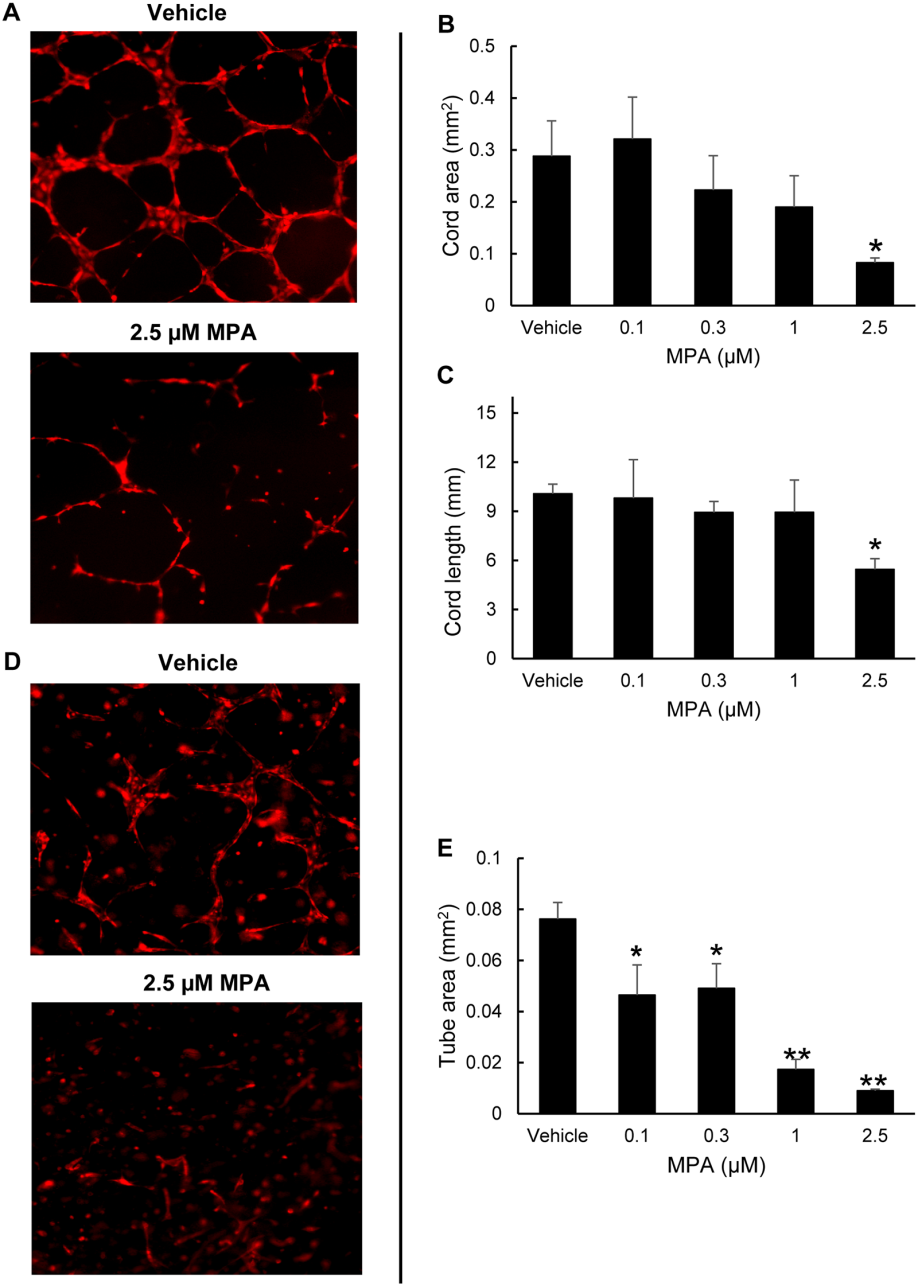
Diminished ECFC vasculogenic function after MPA treatment. Left panel shows representative photomicrographs (magnification, 5x) of TdTomato ECFC following no treatment (vehicle) or treatment with 2.5 μM MPA in 2D assay (A) and 3D assay (D). 2D Matrigel assay showed that there is decreased average total cord length (C) and cord area (B) after MPA treatment. 3D collagen assay showed decreased total vessel area (E) after MPA treatment. *P <0.05, **P <0.005 compared to vehicle (n = 3). Abbreviations: ECFC = endothelial colony forming cells, 2D = two-dimensional, 3D = three-dimensional, EGM = endothelial growth medium, MPA = mycophenolic acid, mm = millimeter.

Normal endothelial cell reparative function is also important in maintaining adequate vascular homeostasis. Therefore, we looked at the effect of MPA on ECFC vasculogenesis using 2D Matrigel and 3D collagen gel assays. In the 2D assay, cells align with each other and form cord networks. The addition of MPA 2.5 μM concentration significantly reduced average cord area and total cord length by 29% (0.082 mm^2^±0.009, P = 0.037) and 54% (5.45 mm±0.75, P = 0.026) respectively, compared to untreated cells (Fig 4A, 4B and 4C). In the 3D assay, single cells are suspended in gel and form vascular structures that emerge through a process of cytoplasmic vacuolation [20]. The 3D assay is thus likely to be more representative of vessel formation *in vivo* than the 2D assay. The capillary-like structures with patent lumens were quantified. Treatment with 1 μM MPA concentration showed very rudimentary tubular structures (Fig 4D). The average vascular area of cells exposed to MPA 1 μM and 2.5 μM concentrations was significantly smaller than the untreated cells by 78% (0.017 mm^2^±0.004, P = 0.0006) and 88% (0.009 mm^2^±0.0006, P = 0.003) respectively, compared to the untreated cells (Fig 4E). These data suggest that MPA inhibits the functional vasculogenic properties of ECFC.

### Cell cycle

MPA causes cell cycle arrest in activated T and B lymphocytes in the G_1_ phase [21]; therefore we examined the effect of MPA on EC cycle progression using BrdU assay and PI staining. BrdU incorporation into the DNA of ECFC was used to detect changes in the percentage of cells in the G_1_, S and G_2_ phase of the cell cycle. Cells incubated with MPA 1 μM resulted in a significant increase in G1 phase and significant decrease in S phase as compared to the vehicle control (S2 Fig). With PI staining, we found a higher percentage of cells in the G_0_/G_1_ phase and consequent lower percentage of cells in the S phase after 3 (G_0_/G_1_: 91.9% vs. 82.5% in complete EGM-2 medium; S: 3.6% vs. 6.5% in complete EGM-2 medium) and 7 days (G_0_/G_1_: 92% vs. 65.6% in complete EGM-2 medium; S: 3.8% vs. 13.6% in complete EGM-2 medium) of exposure to MPA 1μM (S3 Fig). This result suggests that MPA treatment causes ECFC cell cycle arrest by blocking G_0_/G_1_ to S phase transition.

### MPA does not cause apoptosis

To investigate whether the effect observed on cell proliferation was due to cell death, we performed an apoptosis assay using flow cytometry. The percentage of annexin V–negative/PI–negative (viable cell), annexin V-positive (undergoing early death) and PI-positive (dead or necrotic cells) cells cultured with or without 1 μM MPA were not statistically different (S4 Fig). Our results indicate that the inhibition of proliferation after MPA treatment was not due to enhanced cell death.

### MPA induces premature senescence of ECFC via p21/p53 pathway

**Fig 5:**
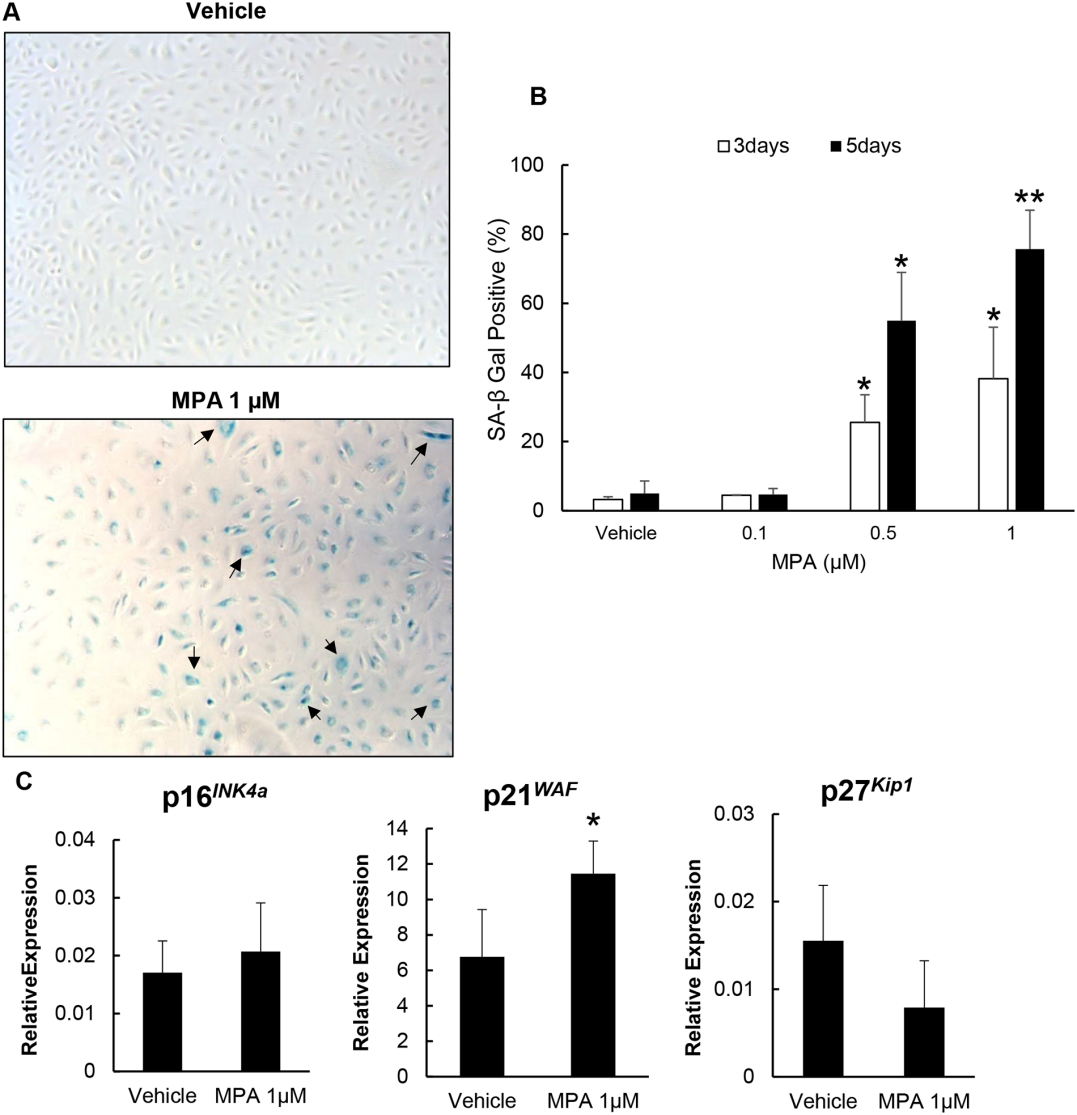
MPA induced ECFC senescence. MPA treated cells have increased SA-β-gal staining and higher senescence related p21^WAF^ gene expression. (A) Photomicrograph of SA-β-gal staining of ECFC cultured with vehicle and 1 μM for 5 days. Blue color (arrow) indicates cellular senescence. (B) Quantification of SA-β-gal staining. At least 500 cells per individual sample were counted. The number of SA-β-Gal-positive cells treated with 0.5 and 1μM MPA concentrations were significantly greater than untreated cells. (C) Gene expression of p16^INK4A^, p21^WAF^ and p27^Kip1^ in untreated and treated ECFC normalized to B2M products. Results represent the mean ± SD compared (n = 3). *P < 0.05, **P <0.001 relative to vehicle control. Abbreviations: MPA = mycophenolic acid, SA-β-gal = senescence associated-beta galactosidase, ECFC = endothelial colony forming cell, B2M = beta-2-microglobulin.

Senescence is another mechanism by which cell growth and proliferation is inhibited, thus we wanted to determine whether MPA induces senescence in ECFC using SA-β-gal staining. We observed a significant dose-dependent increase in SA-β-gal positive cells (8-fold and 12-fold after 3 days of treatment with 0.5 μM and 1 μM MPA, respectively). This effect was more pronounced after 5 days of MPA treatment (Fig 5A and 5B). These results suggest that MPA drives ECFC into a state of senescence.

**Fig 6:**
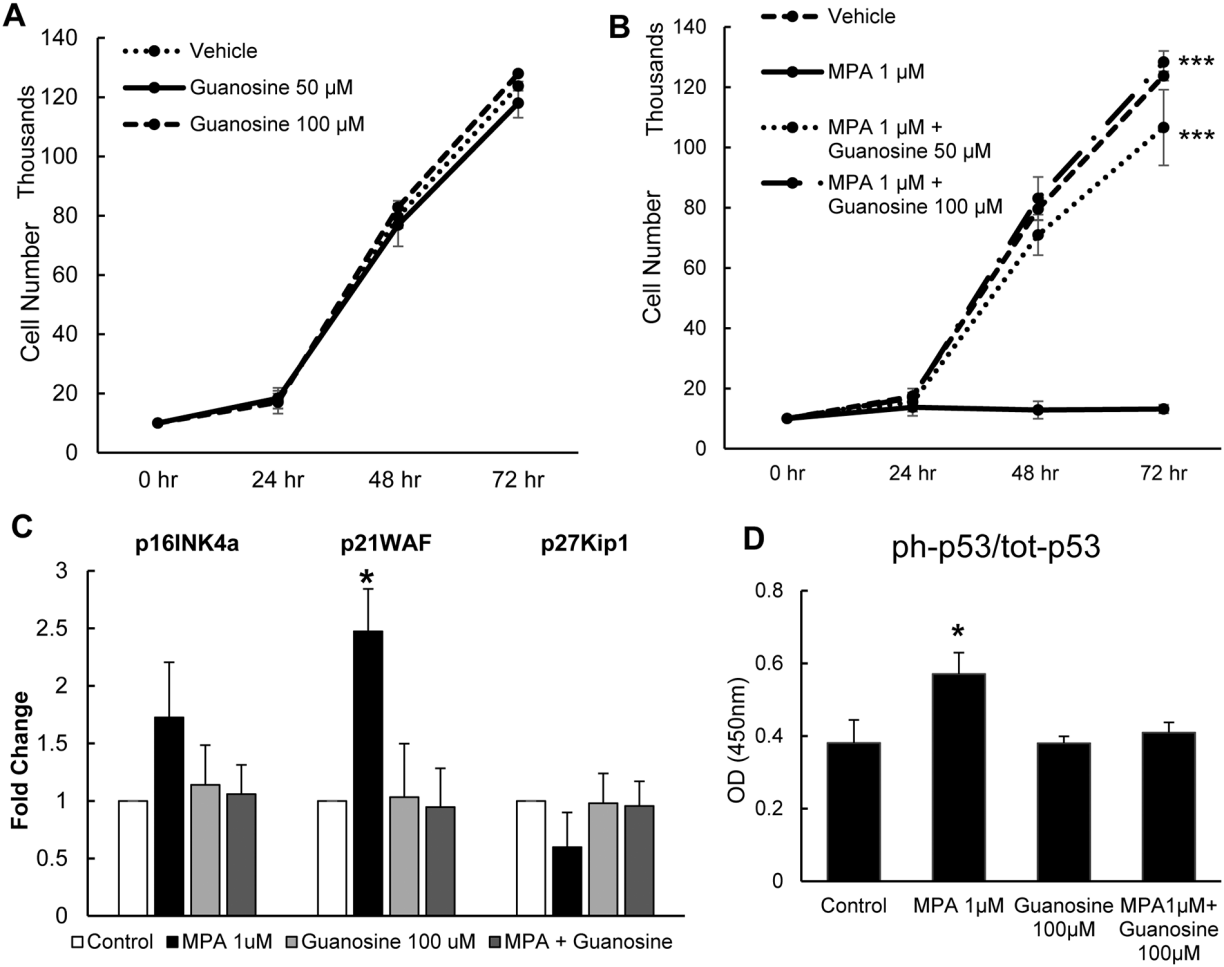
Guanosine reversed MPA’s inhibitory effect on cell proliferation and senescence markers. Cell proliferation using trypan blue exclusion assay shows that guanosine is not cytostatic or inhibitory (A), and that addition of guanosine rescues ECFC proliferation in a dose dependent manner (B). Relative fold expression of senescence associated gene markers p16^INK4a^, p21^WAF^ and p27^KiP1^(C). Phosphorylation of p53 normalized to total p53 level in cell lysates (D). Results represent the mean ± SD compared (n = 3). *P < 0.05, ***P <0.001 relative to vehicle. Abbreviation: MPA = mycophenolic acid, ECFC = endothelial colony forming cell, OD = optical density, ph-p53 = phosphorylated p53, tot-p53 = total p53.

To identify potential mechanism by which MPA enhances susceptibility of ECFC to undergo senescence, we determined the expression of p16^*INK4a*^, p21^*WAF*^and p27^*Kip1*^, cyclin-dependent kinase (CDK) inhibitors and known senescence-associated inducers of cell cycle arrest. The expression of p21^*WAF*^ in cells exposed to 1 μM MPA (11.45±1.84) increased significantly compared to untreated cells (6.77±2.67), but no significant difference in p16^*INK4a*^ and p27^*Kip1*^ expression (Fig 5C) was found in these groups. Since p53 directly induces p21^*WAF*^ and is a key regulatory component of stress-inducible cell growth arrest, we assessed whether MPA-treated cells displayed increased p53 activation. By comparing the ratio of UV absorbance between phosphorylated (ph)-p53 and total (tot)-p53, we observed a significantly higher level of p53 phosphorylation (activated state) in the MPA treated cells compared to controls (Fig 6D). Altogether, these results show that MPA-treated cells become highly senescent, and imply that p21^*WAF*^ gene upregulation is dependent on p53 activation to drive ECFC into senescence as a result of MPA treatment.

### Guanosine restores ECFC proliferation and senescence

The consequence of IMPDH inhibition by MPA is a reduction in guanine nucleotide pools [21]. We tested whether MPA exerts its effect on ECFC IMPDH enzyme activity by measuring whether the addition of guanosine can reverse the suppressive cell growth effects of MPA. Guanosine at 50 or 100 μM alone was neither mitogenic nor inhibited cell growth (Fig 6A). 100 μM guanosine together with 1μM MPA completely restored ECFC proliferation after 48 hours and 72 hours. We observed similar effects in cells exposed to medium containing 50 μM guanosine and 1 μM MPA but cell proliferation rescue was 17% lower (NS) than 100 μM of guanosine (Fig 6B). Thus, guanosine supplementation was sufficient to rescue ECFC from the anti-proliferative effects of MPA in a dose dependent fashion, consistent with the known mechanism of action of MPA on cells.

Based on the ability of guanosine to rescue ECFC proliferation, we tested whether guanosine would rescue the cells from senescence. The addition of 100 μM guanosine decreased p21^*WAF*^ gene expression to levels that were similar to those seen in control cells (Fig 6C). Accordingly, the level of p53 activation of cells exposed to both MPA and guanosine was not significantly different from the control cells (Fig 6D). These results imply that the cytostatic effect of MPA in ECFC is through guanosine depletion via IMPDH inhibition, and the addition of exogenous guanosine rescues MPA-induced inhibition in cell proliferation and senescence.

## Discussion

In this study, we have shown that MPA has potent anti-angiogenic properties and exerts a dose-dependent inhibition of ECFC proliferation *in vitro* by driving cells towards senescence. MPA treatment severely impairs the ability of ECFC to form blood vessels *in vitro* and diminishes ECFC cellular migration.

Our in vitro results are consistent with previously reported data that MPA/MMF treatment at 1–100 μM concentrations inhibits proliferation and blocks angiogenesis *in vivo* in various types of mature EC [22–24]. However, the direct effect of MPA on endothelial precursor cell (EPC) that have reparative and regenerative potential has not been previously investigated. Results of studies evaluating EPC number and function in patients with CIRD, like systemic lupus erythematosus, systemic scleroderma and primary systemic vasculitis, are inconsistent [25–28]. One reason is because there is no EPC-specific cell markers and hence, difficulty in accurately defining EPC identity. Many studies purportedly examining EPC number and function were in fact analyzing proangiogenic hematopoietic cells, which are circulating bone marrow-derived cells that plays a role in neovascularization of damaged tissue but do not fully integrate as true endothelial cells into the remodeled endothelium [29–32]. Recent work has highlighted the multi-cellular network of proangiogenic cells and vascular endothelial cells required to effectively repair damaged endothelium.[32] Early work from our group showed that ECFC display all features of the originally defined endothelial progenitor cell, and the infusion of ECFC into pre-clinical animal models of hind limb ischemia, myocardial infarction and retinal ischemia enhanced vascular recovery [19, 33–35]. Due to ECFC’s vessel forming potential and importance in maintaining EC homeostasis, the inhibitory effect of MPA observed in this study highlights the concern for vascular off-target toxicity of immunosuppressive agents. Further clinical studies are required to determine first, the intrinsic behavior of ECFC in patients with CIRD and second, whether the drugs exert similar *in vitro* effects on ECFC in rheumatic patients maintained on immunosuppressive therapies. Since these cells are rare in the peripheral circulation [19], analysis will require sampling a high volume of blood from patients which is challenging in the pediatric population. It is important to note that we started with a concentration range of MPA (1–5 μM) that includes clinically relevant and targeted serum trough concentration of 1–3.5 μg/mL (3.3–11.5 μM). The concentration range was reduced by 10-fold in subsequent experiments due to the potent inhibitory effects of MPA on ECFC *in vitro*. This raises concern that MPA at current patient therapeutic doses may possess potential vascular toxicity especially with the notable narrow therapeutic index of MPA and large intra- and inter-individual variation in both MPA plasma concentrations and levels of IMPDH activity [36, 37].

We showed that MPA-treated ECFC display senescent-like features such as lack of proliferation, cell cycle arrest, and increased SA-ß-galactosidase activity. The p53/p21 signaling pathway known to be a critical regulator of cell survival is also activated in a senescence process that subsequently leads to vascular dysfunction [38]. We demonstrated a higher level of expression of the CDK inhibitor p21^*WAF*^ and p53 activation. In the cardiovascular literature, there is accumulating evidence that implicates endothelial cell senescence in the initiation and progression of atherosclerosis. Vascular senescence had been identified in human atherosclerotic lesions and is thought to be an important contributor to the pathogenesis of age-associated conditions like heart failure and diabetes [39–41]. Many adult and childhood-onset systemic rheumatic diseases are complicated by endothelial dysfunction, premature atherosclerosis and increased CV disease that are not fully explained by traditional risk factors and the risks persist even after disease control is clinically attained [2, 5]. Immune related cells undergoing senescence may be a desired effect to control overactive lymphocytes, or unwanted breakdown in self-tolerance favoring the development of autoimmune disease [42–44]. The possibility that senescent changes in EC may contribute to the pathophysiology and increased CV morbidity of rheumatic disease is plausible and has not been fully examined. Oxidative stress, pro-inflammatory cytokines, enhanced telomere shortening with inadequate repair, and the destructive influence of senescence-associated secretory phenotype are some of the hypothesized mechanistic processes potentially involved [45–47]. It is beyond the scope of this study to investigate the specific regulatory mechanisms and pathways that occur after MPA treatment of ECFC. However, it will be an interesting area to pursue in future research. In our work, MPA caused senescence without induction of apoptosis in ECFC, which is in contrast to what has been observed in T cells [48–49].

We added guanosine to assess for IMPDH independent GTP synthesis indirectly. Exogenous guanosine supplementation restored ECFC proliferation and interestingly, it reversed the senescent effect of MPA at a transcriptional and functional level. This finding proves that despite the ability of EC to scavenge free purine and/or purine-containing ribonucleosides and deoxyribonucleosides to maintain critical cellular levels of these essential metabolites [50], the guanine salvage pathway is unable to compensate for the low GTP pool in order to prevent cell growth arrest in MPA treated cells.

In conclusion, our study demonstrates that MPA has a potent inhibitory and anti-angiogenic effect on EC. Here, we provided a plausible molecular mechanism in the senescence pathway not previously demonstrated. MPA drives ECFC to go into premature senescence and thus is likely to have pathophysiologic significance in the early atherosclerosis seen in CIRD. The direct short- and long-term clinical consequence of these effects on cardiovascular health and morbidity are unknown. This warrants a better understanding of how immune suppressive drugs disrupt vascular homeostasis and contributes to endothelial dysfunction. In the clinic, MMF and MPA are favored by many rheumatologists due to their steroid-sparing properties, reversible side effect profile, ease of administration and renal protective effects [51–53]. However, recognition and understanding the mechanism of this toxicity will be crucial for optimal patient management. This study highlights the need for more translational research to gain insight into mechanistic link among endothelial cell biology, systemic inflammation, and vascular injury and repair in patients with rheumatic disorders. Further knowledge can usher the discovery of better and safer immunosuppressive therapies and drug regimens in treating patients with systemic rheumatic disease that optimize desired effects while minimizing unwanted drug toxicities, the basis for individualized therapy.

## Supporting information

S1 Fig 2D Matrigel and 3D collagen assay. (PDF)

S2 Fig Cell cycle analysis using BrdU and 7-AAD staining. (PDF)

S3 Fig Block in G_0_/G_1_ to S phase transition after MPA treatment. (PDF)

S4 Fig No effect on apoptosis after adding MPA. (PDF)

S1 Table Primers used in senescence. (PDF)
