# Supplementary material for: Mycophenolic acid induces senescence of vascular precursor cells
Source: PLoS One. 2018 Mar 14;13(3):e0193749. doi: 10.1371/journal.pone.0193749 (PMC5851606; doi:10.1371/journal.pone.0193749)
Supplement: S1 Fig — (PDF) [file pone.0193749.s001.pdf]

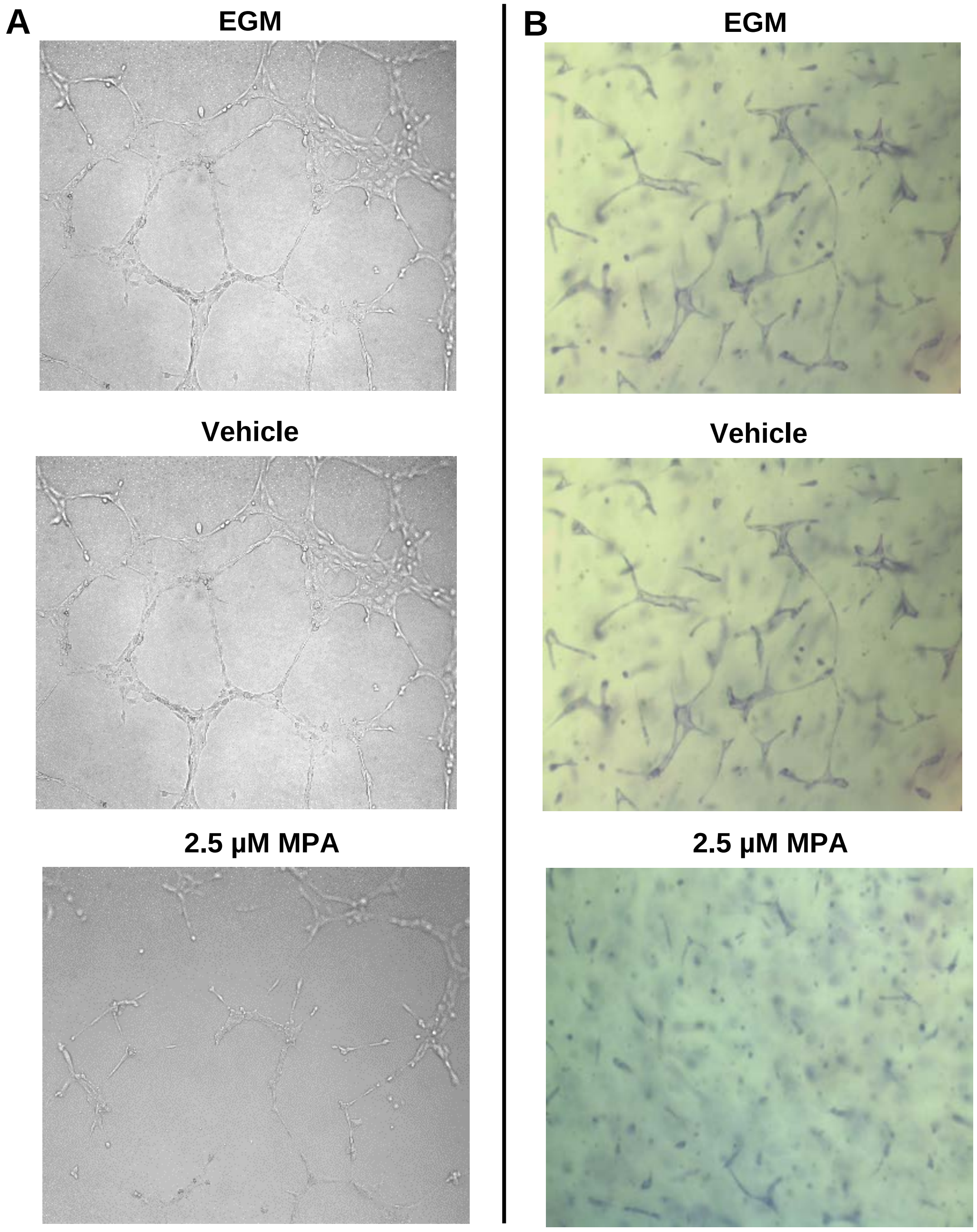

S1 Fig. 2D Matrigel and 3D collagen assay

Representative photomicrographs (magnification, x10) of unlabeled ECFC following exposure to EGM control media, vehicle or 2.5 µM MPA in 2D assay **(A)** and 3D assay **(D).** ECFC function was quantitated using unlabeled ECFC. Abbreviations: ECFC = endothelial colony forming cells, 2D = two-dimensional, 3D = three-dimensional, EGM = endothelial growth medium, MPA = mycophenolic acid, µm = micromolar
