# Supplementary material for: Mycophenolic acid induces senescence of vascular precursor cells
Source: PLoS One. 2018 Mar 14;13(3):e0193749. doi: 10.1371/journal.pone.0193749 (PMC5851606; doi:10.1371/journal.pone.0193749)
Supplement: S2 Fig — (PDF) [file pone.0193749.s002.pdf]

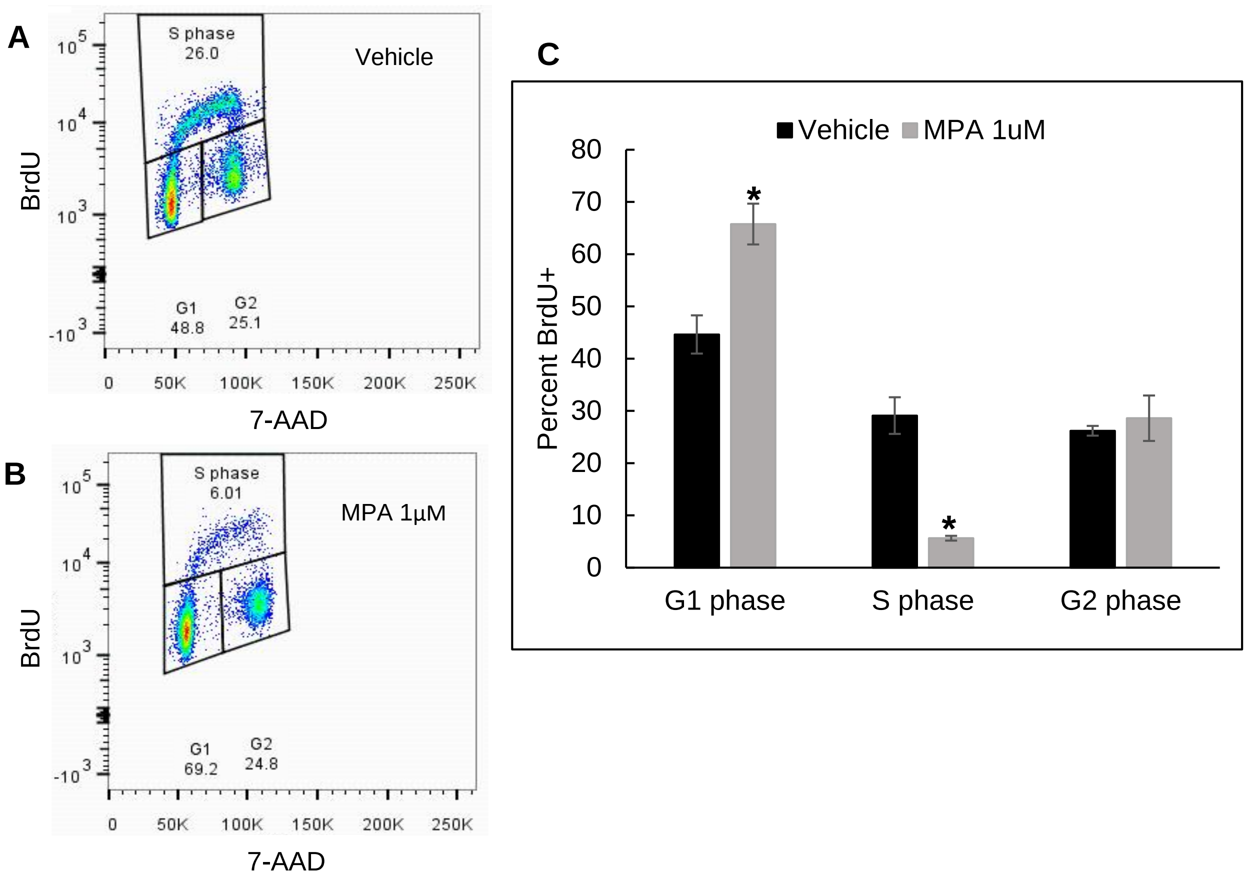

S2 Fig: Cell cycle analysis using BrdU and 7-AAD staining

Representative flow cytometry gating strategy to measure cell cycle progression of ECFC without **(A)** and with **(B)** MPA treatment. Quantitation of cell cycle analysis using flow cytometry data (n=3). *P<0.01. Abbreviations: MPA = mycophenolic acid
