# Supplementary material for: Mycophenolic acid induces senescence of vascular precursor cells
Source: PLoS One. 2018 Mar 14;13(3):e0193749. doi: 10.1371/journal.pone.0193749 (PMC5851606; doi:10.1371/journal.pone.0193749)
Supplement: S3 Fig — (PDF) [file pone.0193749.s003.pdf]

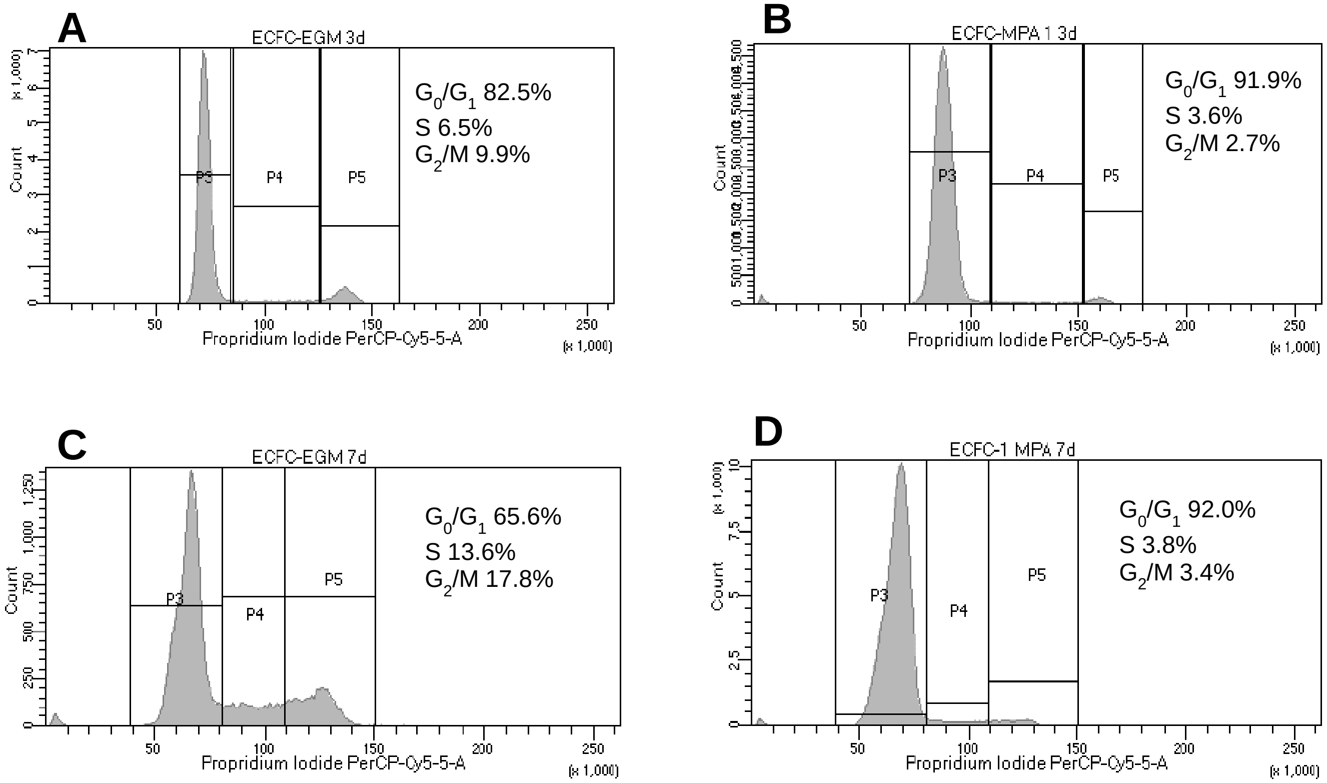

S3 Fig: Block in G_0/G_1 to S phase transition after MPA treatment

Histogram analysis of the subpopulation of cells in cell cycle phases G_0/G_1, S and G_2/M represented as a percentage of the total cell count analyzed on day 3 **(A-B)** and day 7 **(C-D)** of incubation without **(A,C)** and with **(B,D)** MPA. Abbreviations: MPA = mycophenolic acid, ECFC = endothelial colony forming cell, 3d = three days, 7d = seven days
