# Supplementary material for: Mycophenolic acid induces senescence of vascular precursor cells
Source: PLoS One. 2018 Mar 14;13(3):e0193749. doi: 10.1371/journal.pone.0193749 (PMC5851606; doi:10.1371/journal.pone.0193749)
Supplement: S4 Fig — (PDF) [file pone.0193749.s004.pdf]

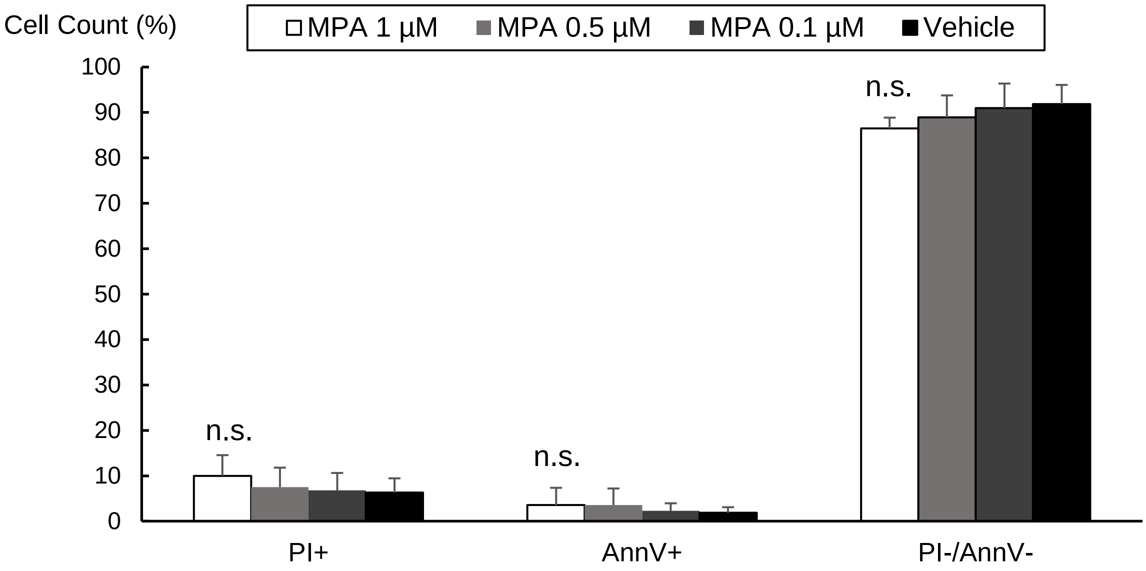

S4 Fig. No effect on apoptosis after adding MPA

Flow cytometric analysis of cells labelled with Annexin-V–PI double staining showing the number of viable (PI-/AnnV-), early death (AnnV+) and apoptotic (PI+) cells. Results expressed as means ± SD (n=3). Abbreviations: MPA = mycophenolic acid, PI = propidium iodide, AnnV = annexin V, n.s. = not significant
