# Supplementary material for: Mycophenolic acid induces senescence of vascular precursor cells
Source: PLoS One. 2018 Mar 14;13(3):e0193749. doi: 10.1371/journal.pone.0193749 (PMC5851606; doi:10.1371/journal.pone.0193749)
Supplement: S1 Table — (PDF) [file pone.0193749.s005.pdf]

Gene	Sequence (5'->3')
Human p16^{INK4a}	Forward: GGGGGCACCAGAGGCAGT
	Reverse: GGTTGTGGCGGGGGCAGTT
Human p21^{WAF}	Forward: AGTCAGTTCCTTGTGGAGCC
	Reverse: GACATGGCGCCTCCTCTG
Human p27^{Kip1}	Forward: TAATTGGGGCTCCGGCTAAC
	Reverse: GAAGAATCGTCGGTTGCAGGT
Human β-2-microglobulin	Forward: CTCCGTGGCCTTAGCTGTG
	Reverse: TTTGGAGTACGCTGGATAGCCT

S1 Table. Primers used in senescence
